# Potential Usefulness of a Wakame/Carob Functional Snack for the Treatment of Several Aspects of Metabolic Syndrome: From In Vitro to In Vivo Studies

**DOI:** 10.3390/md16120512

**Published:** 2018-12-17

**Authors:** Cristina Martínez-Villaluenga, Elena Peñas, Daniel Rico, Ana Belén Martin-Diana, Maria P. Portillo, Maria Teresa Macarulla, Daniel Antonio de Luis, Jonatan Miranda

**Affiliations:** 1 Department of Food Characterization, Quality and Safety, Institute of Food Science, Technology and Nutrition (ICTAN-CSIC), Juan de la Cierva, 3, 28006 Madrid, Spain; c.m.villaluenga@csic.es (C.M.-V.); elenape@ictan.csic.es (E.P.); 2 Department of Research and Technology, Agrarian Technological Institute of Castilla and Leon (ITACyL), Government of Castilla and Leon, Ctra. de Burgos Km. 119, 47071 Valladolid, Spain; ricbarda@itacyl.es (D.R.); mardiaan@itacyl.es (A.B.M.-D.); 3 Nutrition and Obesity Group, Department of Nutrition and Food Science, Faculty of Pharmacy, University of Basque Country (UPV/EHU) and Lucio Lascaray Research Center, 01006 Vitoria, Spain; mariapuy.portillo@ehu.eus (M.P.P.); mariateresa.macarulla@ehu.eus (M.T.M.); 4 CIBEROBN Physiopathology of Obesity and Nutrition, Institute of Health Carlos III (ISCIII), 28029 Madrid, Spain; 5 Endocrinology and Nutrition Department, Hospital Clínico Universitario de Valladolid-IEN, Facultad de Medicina, Universidad de Valladolid, 47005 Valladolid, Spain; dadluis@yahoo.es

**Keywords:** metabolic syndrome, rats, functional snack, in vitro, wakame, carob

## Abstract

Metabolic syndrome (MetS) greatly increases the risk of cardiovascular diseases and type 2 diabetes mellitus. The aim of this study was to evaluate the efficacy of functional snacks containing a combination of wakame (W) and carob pod (CP) flours in reducing markers associated with MetS. The mechanisms of action underlying these effects were also evaluated. In vitro approaches were carried out in mature 3T3-L1 adipocytes and RAW 264.7 macrophages treated with different doses of extracts from W, CP, or a combination of both. Furthermore, an in vivo experiment was conducted in rats with MetS treated with normal-caloric diets containing different snack formulations with combinations of 1/50 (snack A) or 1/5 of wakame/carob (snack B). In vitro experiments results indicated that both W and CP had delipidating effects, but only the latter induced anti-inflammatory and anti-hypertensive effects. As far as the in vivo study is concerned, snack B was ineffective and snack A showed an anti-hypertensive effect in rats with MetS. The present study shows for the first time the in vitro efficacy of a W and CP combination as an anti-inflammatory, delipidating, and anti-hypertensive tool, and its potential usefulness in treating MetS.

## 1. Introduction

Metabolic syndrome (MetS) is a cluster of risk factors associated to cardiovascular disease and type 2 diabetes mellitus (T2DM), mainly suffered by Western populations [1]. According to the clinical classification of the National Cholesterol Education Program Adult Treatment Panel IIII [2], the five factors (among which at least three must be followed) for the definition of MetS are: hypertriglyceridemia, waist circumference increase, low HDL-cholesterol, high blood pressure, and fasting hyperglycemia. Among the causes that exacerbate this syndrome are age, genetic conditions, and an inadequate lifestyle, including a sedentary lifestyle and the consumption of high-calorie foods, rich in carbohydrates, saturated fats, and salt [3].

Hypotheses about the etiology of MetS consider the chronic state of the inflammatory pattern as the starting point, since this may degenerate to insulin resistance and/or cardiovascular diseases [4]. It has also been observed that adipose tissue plays a role in some pathophysiological mechanisms involved in the development of dyslipidemia, insulin resistance, and T2DM. Thus, today it is recognized as a key tissue of insulin resistance and that the MetS is related to it [5]. White adipose tissue (WAT) mass is defined by the size and number of adipocytes. Consequently, metabolic processes occurring inside adipocytes such as de novo lipogenesis, lipolysis, glucose uptake, triglyceride synthesis, fatty acid oxidation, apoptosis, or adipogenesis are essential to determine fat mass [6].

Due to the side effects associated with currently available drugs, as well as their limited efficacy [7], in recent decades, the potential of natural products in combating MetS has aroused great interest in scientific research [8]. Natural products from plant and marine sources, including their isolated extracts or bioactive components, have been demonstrated to be effective in the prevention and treatment of MetS and its co-morbidities [9,10]. Brown algae (*Phaeophyceae*), rich in chlorophylls, sterols, carotenoids, and polyunsaturated fatty acids, have shown positive effects on health, such as anti-hyperglycemic activity, antioxidant properties, and anti-obesity capacity, in animal models [11]. These algae are regularly consumed in Asian countries, such as Korea or Japan. Among brown algae, the *Undaria pinnatifida* (Wakame) is one of the most important. The major components of *U. pinnatifida* include carbohydrates, such as monosaccharides, polysaccharides (including sacran, mannan, and xylan), and dietary fiber; in lower proportion it contains lipids, proteins, vitamins, amino acids, and its derivatives, such as methacrylic acid and tauric acid, minerals, as well as some phytochemicals, including flavonoids, alkaloids, and sterols [12,13,14]. There are numerous animal studies that support its potential use against obesity, dyslipidemia, inflammation, and insulin resistance [15,16]. Results found by our group, also highlight the wakame (W), among other algae, for its anti-inflammatory, anti-hypertensive, and fat-lowering effects [17].

As in the case of algae, pulses are also capturing researchers’ attention due to their healthy properties [18]. This is especially true of the carob tree (*Ceratonia siliqua* L.). Many studies confirm that the fruit of the carob tree may have benefits on human health and may be used both as a preventive or therapeutic agent for certain chronic diseases, and is of great interest for the prevention and management of diabetes or hyperlipidemia [19].

Carob fruit is mainly composed of sugars, including sucrose, glucose, and fructose (48–56%), fibers such as cellulose, hemicellulose, and lignin (30–40% in carob’s pulp), gum (85% of carob seed is composed of galactomannan), amino acids, and minerals (mainly potassium and calcium). Furthermore, it also contains phenolic compound. The concentration of compounds in carob fruit ranges from 45–5376 mg gallic acid equivalents 100 g^−1^ and depends on genetic and environmental factors [19]; the main groups are phenolic acids, hydrolysable, and condensed tannins [20].

The potential use of carob pod (CP) and W in the prevention or treatment of human MetS can be addressed through different strategies; in this regard a simple approach would be to include it in the diet as a functional food. Basic research on bioactive ingredients has some limitations, dose being one of the most important. Sometimes doses used for bioactive components, both in vitro and with experimental animals, are very high in comparison with clinical trials or with levels achieved according to bio-availability. In fact, the low concentration of W bioactive compounds included in the diet could be the reason that explains the lack of body fat reduction in some studies [21] compared to others where the effect was observed [15]. In this context, in recent years, one of the strategies most used in the development of functional foods is the combination of functional ingredients [22]. In the present research, the efficacy of new functional snacks based on the combination of W and CP flour for MetS treatment was evaluated. The mechanisms of action underlying these effects observed by the combination of W and CP were also analyzed.

## 2. Results

### 2.1. In Vitro Fat-Lowering Effect Evaluation and Anti-Hypertensive Activity of W, CP, and Their Combined Extracts

**Figure 1:**
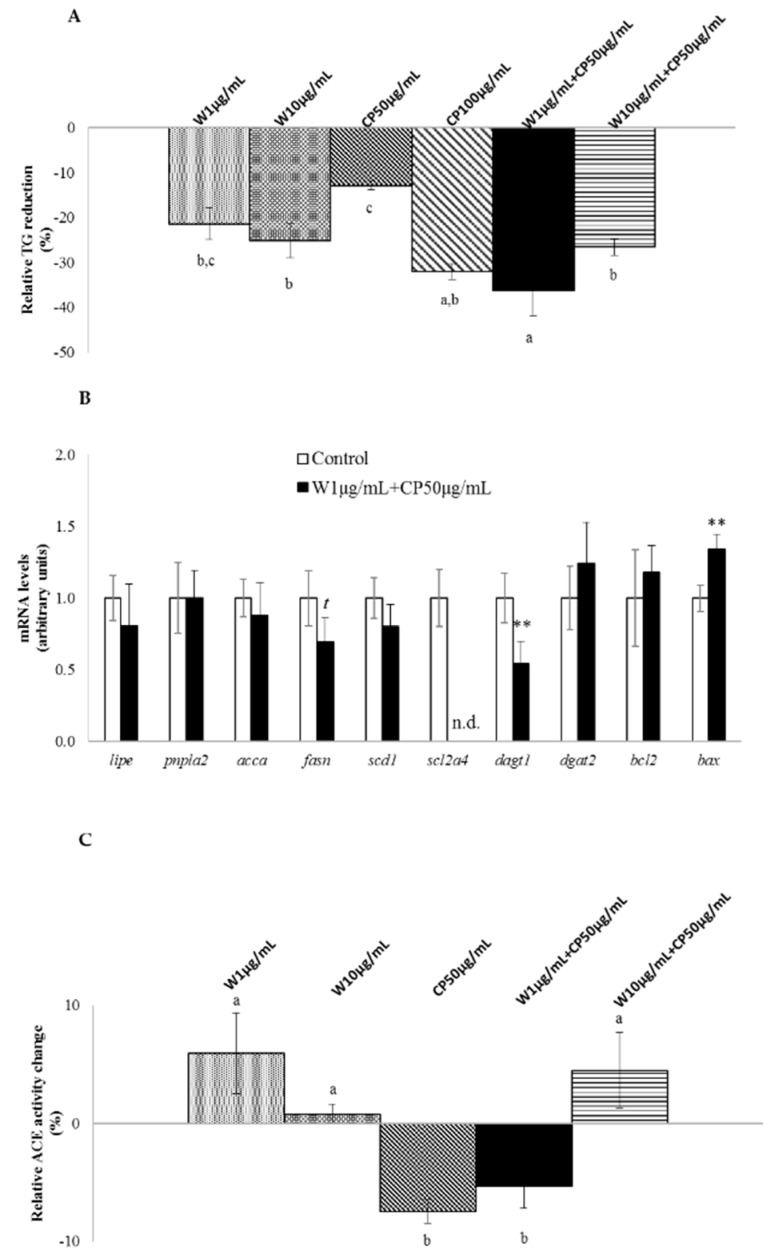
In vitro fat-lowering effect, adipose tissue gene expressions, and anti-hypertensive activity of wakame (W), carob pod (CP), and its combination (W+CP). (**A**) Mature 3T3-L1 adipocytes were treated with 95% ethanol (control) or W (1 or 10 µg/mL), CP (50 or 100 µg/mL), and W+CP (1 µg/mL + CP 50 µg/mL or 10 µg/mL + CP 50 µg/mL) extracts diluted in 95% ethanol for 24 h, and TG content was analyzed. Ethanol concentration in the medium never exceeded 0.1%. Values of three independent experiments carried out in sextuplicate are expressed as mean ± SEM of the reduction percentage compared with the control group (*Y*-axis). Data not sharing a common letter are significantly different (*p* < 0.01). (**B**) mRNA levels of genes related to de novo lipogenesis, glucose uptake, apoptosis, and TG assembly were measured. Data are presented as fold change mean ± SEM of three independent experiments carried out in sextuplicate, *t* < 0.1, ** p < 0.01, n.d.: not detected. (**C**) Angiotensin-converting enzyme (ACE) inhibitory activity was evaluated. Values of three independent experiments carried out in triplicate are expressed as a percentage mean of activity change ± SEM. Data not sharing a common letter are significantly different (*p* < 0.01).

With the exception of CP at 50 µg/mL, all the tested extracts demonstrated significant delipidating effect in 3T3-L1 mature adipocytes (Figure 1A). W extracts reduced triglycerides (TG) content by 21% and 25% at 1 µg/mL and 10 µg/mL doses, respectively. TG decrease reached 32% with CP (100 µg/mL), and 27% with a W 10 µg/mL + CP 50 µg/mL combination. It should be highlighted that the combination of CP (50 µg/mL) with W (1 µg/mL) extract produced the maximum reduction observed (−36%).

With regard to in vitro anti-hypertensive activity, angiotensin-converting enzyme (ACE) inhibitory activity, no significant effect was observed for the W extracts tested. By contrast, the lowest dose of CP extract and its combination with W (W 1 µg/mL + CP 50 µg/mL) revealed significant reductions in ACE activity (Figure 1C).

### 2.2. Gene Expression, Post-Transcriptional Lipolysis, and de Novo Lipogenesis Evaluation in Mature Adipocytes Treated with W and CP Combined Extracts

In Figure 1B the expression of genes related to de novo lipogenesis, glucose uptake, apoptosis, and TG assembly is shown. Mature adipocytes treated with the combination W 1 µg/mL + CP 50 µg/mL showed a significant reduction in the expression *diacylglycerol o-acyltransferase 1* (*dgat1*), solute carrier family 2 member 4 (*scl2a4*) and a significant increase in *BCL2-associated X protein* (*bax*), when compared with the control cells. In addition, a tendency towards reduced values was found in the expression of *fatty acid synthase* (*fasn*).

**Figure 2:**
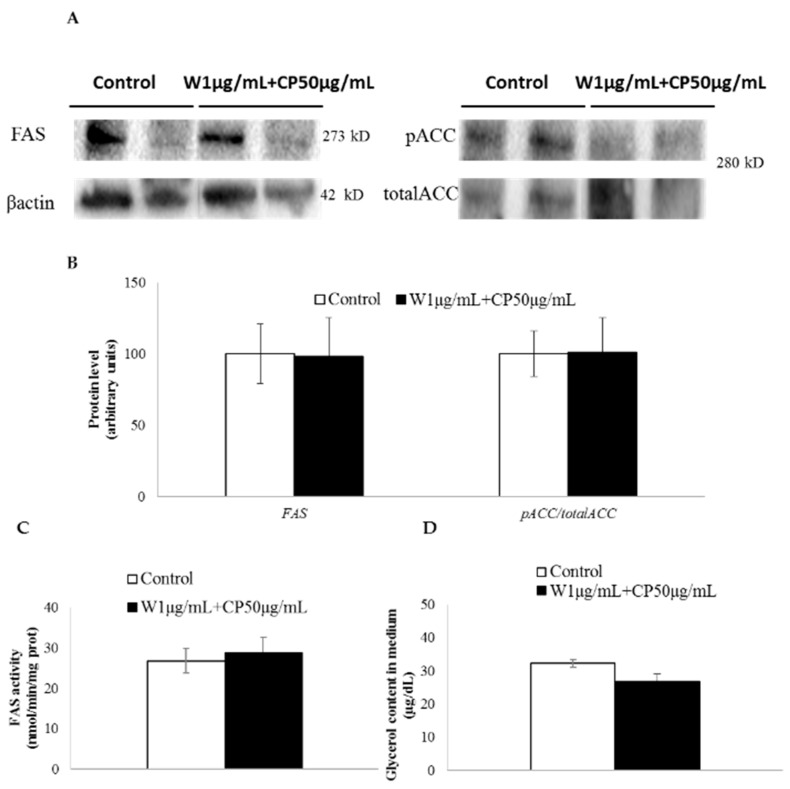
In vitro effect of wakame (W), carob pod (CP), and its combination (W+CP) on the activity of enzymes involved in de novo lipogenesis and glycerol release. (**A**) Mature 3T3-L1 adipocytes were incubated with 95% ethanol (control) or W 1 µg/mL + CP 50 µg/mL extracts diluted in 95% ethanol for 24 h, and Western blotting was used to determine the cleaved FAS, phosphorylated ACC (serine 79), and total ACC protein expression. (**B**) β-actin was used as a loading control, and the quantified expression levels of three independent experiments carried out in duplicates (percentage mean ± SD) by Image software were plotted in the bar graphs. FAS activity (**C**) and glycerol release (**D**) to the medium were determined using spectrophotometry. Values of the three independent experiments carried out in duplicate are expressed as mean ± SEM.

The ratio acetyl-CoA carboxylase (ACC) phosphorylated/total ACC, an index of ACC activity, was not modified after treatment with W and CP combination (Figure 2A). Similarly, no change for FAS protein levels (Figure 2A) or FAS activity (Figure 2B) was detected. Glycerol release to the medium did not vary after W 1 µg/mL + CP 50 µg/mL treatment (Figure 2C).

### 2.3. In Vitro Anti-Inflammatory of W, CP, and Their Combined Extracts

**Figure 3:**
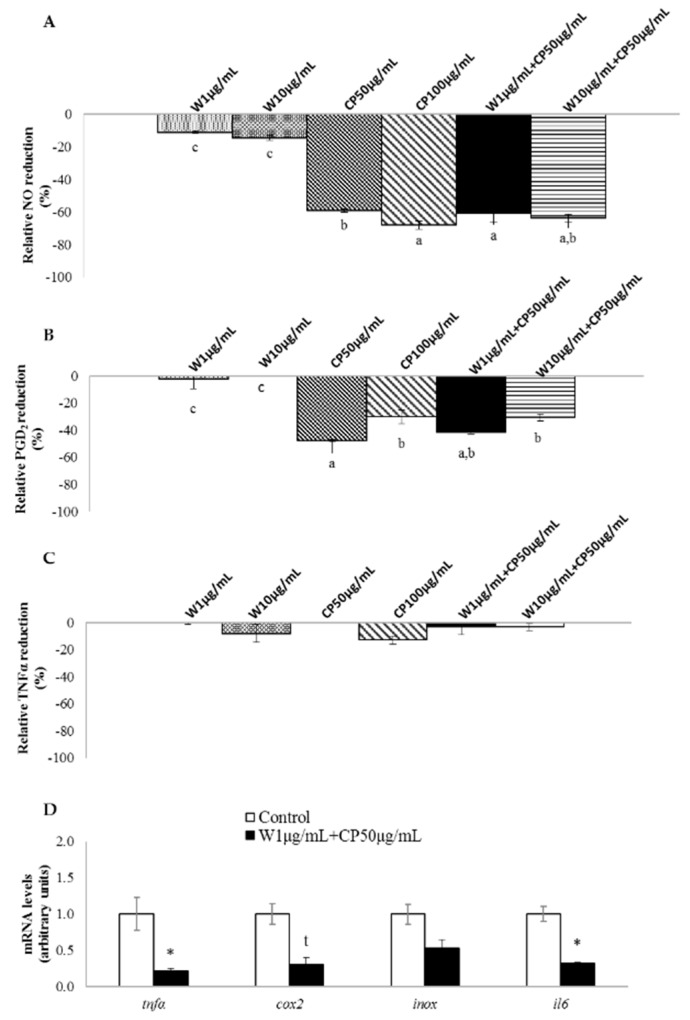
In vitro anti-inflammatory effects of wakame (W), carob pod (CP), and their combinations (W+CP). RAW 264.7 macrophages were treated with serum free medium (control) or W (1 or 10 µg/mL), CP (50 or 100 µg/mL), and W+CP (1 µg/mL + CP 50 µg/mL or 10 µg/mL + CP 50 µg/mL) extracts diluted in serum-free medium for 24 h and then stimulated with LPS. NO (**A**), PGD_2_ (**B**), and TNFα (**C**) release to the medium was analyzed. Values of one independent experiment carried out in sextuplicate are expressed as mean ± SEM of the reduction percentage compared with the control group (*Y*-axis). The letter “c” represents values that are not significantly lower than those of the control group. Data not sharing a common letter are significantly different (*p* < 0.01). (**D**) mRNA levels of genes related to inflammation were measured. Data of one independent experiment carried out with six replicates are presented as a fold change mean ± SEM; *t* < 0.1, * *p* < 0.05.

In RAW 264.7 macrophages, W extracts only reduced the production of nitric oxide (NO) without a dose-response effect. The extracts of CP, as well as both combinations, reduced the production of NO and prostaglandin D_2_ (PGD_2_) (Figure 3A,B). Tested extracts seemed to be ineffective on tumor necrosis factor α (TNFα) reduction (Figure 3C). Marked differences were observed between W extracts and the others. While the NO reduction for both W extracts was around −10%, this reached −60% for CP (50 µg/mL) and the combination of extracts (W 10 µg/mL + CP 50 µg/mL, W 1 µg/mL + CP 50 µg/mL). In this line, a direct comparison between W and CP at the same dose, 10 µg/mL, revealed higher NO-lowering effect for CP (−15%) than for W (−51%).

### 2.4. Gene Expression of RAW 264.7 Cells Treated with W and CP Combination

Lipopolysaccharide-stimulated RAW 264.7 macrophages treated with W 1 µg/mL + CP 50 µg/mL reduced the expression of *tnfα* and *interleukin 6* (*il6*) mRNA levels. Moreover, a tendency towards reduced values was observed for *cyclooxygenase 2* (*cox2*) gene expression (Figure 3D).

### 2.5. Composition of Functional Snacks

The composition of snacks was evaluated. As expected, total carbohydrates and fiber content were the major components: 43.5 and 22.9 g per 100 g of snack, respectively, in the case of snack A, and 43.7, and 22.0 g 100 g^−1^, respectively, in the case of snack B. Fat level was 16.0 g 100 g^−1^ for 1:50 (W:CP) formulation and 16.1 g 100 g^−1^ for 1:5 formulation, meanwhile protein, water and ashes were the minor components (11.9, 4.2, and 1.4 g 100 g^−1^, respectively in the case of snack A, and 12.2, 4.3, and 1.6 g 100 g^−1^, respectively, in the case of snack B).

### 2.6. Food/Energy/Liquid Intake, Total Body Weights, White Adipose Tissue Weights, Interscapular Brown Adipose Tissue Weights, and Gastrocnemius Muscle Weights in Rats Treated with Functional Snacks

No significant differences were observed among groups in final body weight, WAT weights, interscapular brown adipose tissue weight, or gastrocnemius muscle weight (Table 1). Rats treated with control diets (wheat control group -WC- and wheat-oat control group -OC-), as well as with diets including functional snack (the snack A low-dose group -SAL-, the snack A high-dose group -SAH-, and the snack B high-dose group -SBH-), showed lower water intake than MetS rats. No differences in food intake or energy intake were observed between the experimental diets used to treat MetS (WC, OC, SAL, SAH, and SBH) (Table 1).

### 2.7. Blood Pressure and Renal ACE Activity in Rats Treated with Functional Snacks

**Figure 4:**
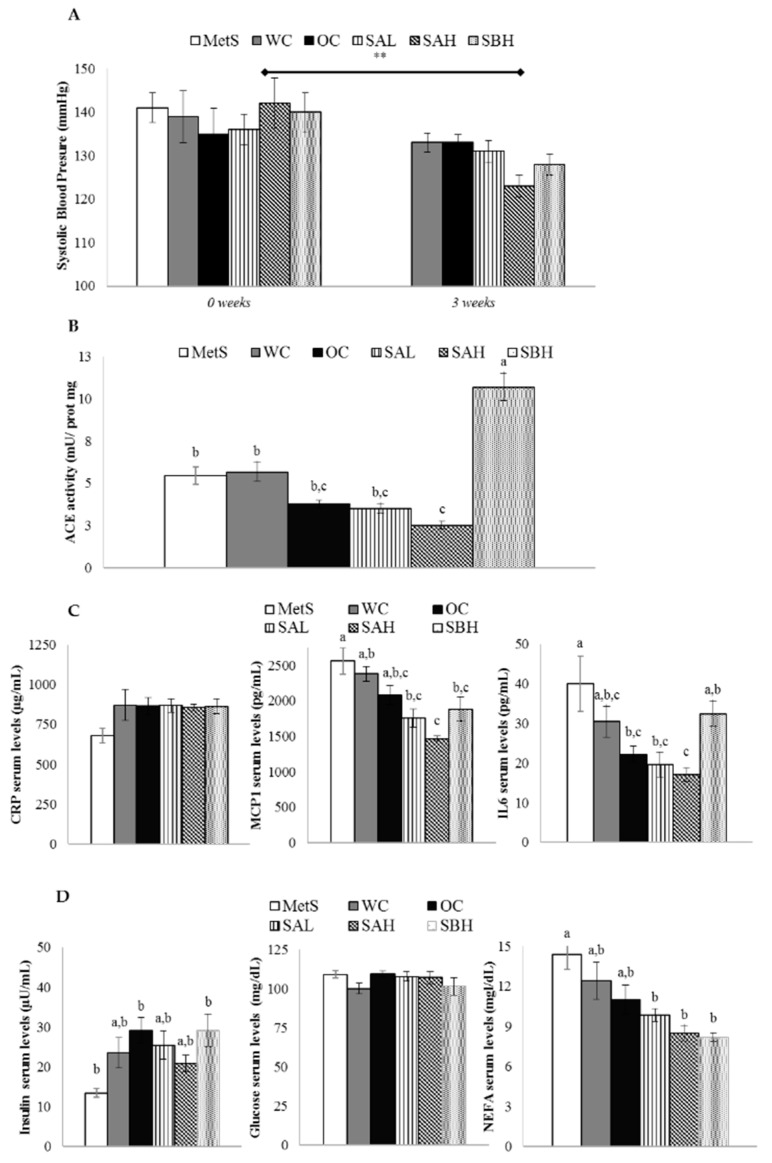
Anti-hypertensive, anti-inflammatory, and glycemic-controlling effects of functional-diet feeding. (**A**) Systolic blood pressure at the beginning of the experiment and after 3 weeks of treatment; (**B**) angiotensin-converting enzyme (ACE) activity in rat kidney at the end of the experiment; (**C**) serum levels of C-reactive protein (CRP), monocyte chemoattractant protein 1 (MCP1), and interleukin 6 (IL6) at the end of the experiment; and (**D**) serum levels of insulin, glucose, and non-esterified fatty acids (NEFA) at the end of the experiment in rats fed a wheat control diet (WC, *n* = 10), a wheat or oat control diet (OC, *n* = 10), a low dose snack A diet (SAL, *n* = 10), a high dose snack A diet (SAH, *n* = 10), and a high dose snack B diet (SBH, *n* = 10) diets. Values are expressed as mean ± SEM. Data not sharing a common letter are significantly different (*p* < 0.05).

At the beginning of the experiment (week 0 of treatment), when MetS was developed, systolic blood pressure (SBP) and diastolic blood pressure (DBP) values were 139 ± 2 and 83 ± 2 Hg mm, respectively. After the treatment, only the SAH group showed a significant decrease of SBP (*p* < 0.01) (Figure 4A). No change in DBP was observed among different groups of rats (data not shown). Rats treated with high doses of functional snacks modified ACE activity in kidneys compared with MetS groups; more specifically, whereas the snack A reduced this enzymatic activity (*p* < 0.05), snack B increased it (*p* < 0.01) (Figure 4B).

### 2.8. Anti-Inflammatory Activity in Rats Treated with Functional Snacks

No differences in C-reactive protein (CRP) serum levels were found among groups of rats (Figure 4C). Rats fed the functional snacks (SAL, SAH, and SBH) showed lower monocyte chemotactic protein 1 (MCP1) serum levels than MetS rats (Figure 4C). The most remarkable decrease was observed in the SAH group, reaching statistical significance when compared with the WC group. In the case of IL6 serum levels, SAH treatment produced the most prominent decrease compared to the MetS group (a 57% reduction). By contrast, SBH diet did not induce a significant modification (Figure 4C).

### 2.9. Functional Snacks Effect on Glycemic Control in MetS Rats

Insulin levels were increased in all expirmental groups in comparison with MetS rats, reaching statistical significance in the case of OC and SBH groups (Figure 4D). Glucose levels were not modified among different groups (Figure 4D). The inclusion of the snacks in the diet (SAL, SAH, and SBH groups) reduced non-esterified fatty acids (NEFA) at the end of the experimental period (Figure 4D).

## 3. Discussion

The present study aimed at analyzing the potential efficacy of new snacks prepared combining W and CP for MetS treatment. Due to the fact that obesity is one factor included in the MetS, the anti-obesity effects of these snacks were assessed. It has been described that obesity is associated with a low-grade inflammatory status. WAT is infiltrated by macrophages, which together with adipocytes, perpetuate a cycle of macrophage recruitment and secretion of free fatty acids deleterious cytokines/chemokines predisposing to develop MetS [23]. Thus, in the present study the potential anti-inflammatory effect of the snacks was also studied.

In order to address the objective proposed, the study followed two different approaches. First, in vitro approach was carried out in 3T3-L1 using mature adipocytes and RAW 264.7 macrophages, where the individual and combined effects of W and CP were evaluated. Second, an in vivo study was performed in MetS rats fed a normal-caloric diet including two types of snacks based on the combination of W and CP. W was chosen from the results of a previous screening study carried out with seven seaweed species, where it was observed that W had the most effective bioactive profile [17]. The dosage used for W and CP was selected on the basis of a previous work, as the lowest dose with a significant effect (submitted data). Furthermore, the results from a sensory-panel study, in which breads, including seaweeds or carob by-products were tested, was taken into account; these experiments demonstrated that the presence of seaweeds reduced sensory properties more than CP [24]. According to cell viability, doses as high as 50 µg/mL of W and CP were considered as safe [17].

In cultured mature adipocytes, W showed a delipidating effect, either at the highest dose or at the lowest dose, which did not show a dose–response pattern. However, in the case of CP, only the high dose was effective. Additive effects were observed when both products were combined at the lowest doses used (W 1 µg/mL + CP 50 µg/mL). By contrast, in the case of the highest doses, no additive or synergistic effects were observed when combined extracts of both products were included in the incubation medium, because their effects were not greater than those induced by CP. These results revealed that with regard to TG reduction, a 1/50 ratio of W/CP, but not 1/5, reached a similar level of decrease (−36%) to that observed in previous studies with W 100 µg/mL (−38%) [17]. As stated in the introduction, one of the most important limitations for functional snack formulation is the dose used. According to the results obtained, W 1 µg/mL + CP 50 µg/mL in vitro treatment was enough to raise the maximum TG-lowering effect. It has been demonstrated that phenolic compounds undergo extensive metabolism after oral ingestion, thus notably reducing their bioavailability, and that only a small amount of them reaches plasma and tissues [25]. This fact, as stated by Langhans in the review about food components in health promotion and disease prevention [26], makes it difficult to extrapolate the positive effects observed in vitro, usually carried out at supraphysiological doses. Therefore, the effective combination of W+CP at low doses (1 and 50 µg/mL, respectively) makes it easier to extrapolate possible uses of these compounds in in vivo studies or in clinical practice.

In order to explain the mechanisms underlying the delipidating effect, the expressions of genes involved in the main metabolic processes of adipocyte triglyceride metabolism were measured in adipocytes cultured with W 1 µg/mL + CP 50 µg/mL. No significant changes were observed in the expression of genes codifying for enzymes involved in lipolysis (*hormone sensitive lipase* and *adipose triglyceride lipase, lipe* and *pnpla* respectively). As far as lipogenic genes are concerned, *acca* and *stearoyl-Coenzyme A desaturase 1* (*scd1*) remained unchanged, but *fasn* showed a tendency toward a reduction. In view of these results, and considering that one of the most active molecules in W, namely fucoxanthin, has been proposed as an antilipogenic molecule [27], we analyzed the protein expression and activity of FAS, and the activity of ACC by measuring the ratio phosphorylated ACC/total ACC, but no changes were observed. Similarly, in order to know whether lipolysis was affected at post-transcriptional level, the amount of glycerol released to the incubation medium was measured, but no significant differences were found. Therefore, it seems that de novo lipogenesis and lipolysis metabolic processes were not involved in the delipidating effect of the analyzed combination.

Moreover, a significant decrease was observed in *dgat2*, and the gene codifying for GLUT4 transporter (*scl2a4*) was not detected. These results showed that the assembly of triglycerides was impaired, not only due to decreases in the activity of a key enzyme of this metabolic process, but also due to the limited availability of glycerol-3P coming from glucose metabolism. Finally, the combination increased the expression of the pro-apoptotic gene *bax* and maintained that of the anti-apoptotic gene *bcl2*, showing that increased apoptosis also contributed to the reduction in total TG accumulated in adipocytes.

Even though the W functional ingredient was enough to promote the TG-lowering effect, it poses a clear limitation with regard to combatting the inflammation. In order to overcome this handicap, CP was also evaluated in the present study. In vitro assays demonstrated that the most clear anti-inflammatory action was found in macrophages treated with CP. It has been proposed that the compounds responsible for this action, by means of their anti-oxidant effects, are phenolic compounds [28], with phenolic acids, gallotannins, and flavonoids being the most significant [19]. As in the case of TG-lowering effect, no additive or synergistic was observed with the combination of CP and W. Nevertheless, taken together the cell culture results demonstrated that the combination of W with CP represents a more complete formulation that is useful for MetS treatment.

The positive results obtained in the in vitro study encouraged us to address an in vivo study. In this experiment, rats were fed a high-fat high-sucrose diet in order to induce MetS, and then they were treated with two snacks elaborated with W and CP in different proportions, as described in the Material and Methods section. The high-dose snack diets evaluated the effect of the complete substitution of complex carbohydrates using functional snacks [29], and the low-dose snack diet simulated a human intervention study, with an affordable functional snack intake (50 g per day, in a total contribution of complex carbohydrates of 250 g) [30]. Surprisingly, the expected anti-obesity effect was not observed because no reductions were found in WAT weights among experimental groups. Although no differences were observed by comparing experimental groups (SAL, SAH, and SBH) with control groups (OC and WC) in terms of energy intake, overall modification of energy expenditure (EE) by treatment cannot be discarded. Some authors have postulated the use of poly(phenols) as a strategy for weight loss, as they can increase EE [31]. In this line, it is important to highlight that W and CP are a good source of these compounds. Similarly to phenolic compounds, fucoxanthin carotenoid, included in W, also increases EE [32]. Unfortunately, an indirect calorimetry was not addressed in the present research, implying a limitation of the study. Wu et al. demonstrated that fucoxanthin supplementation to high-fat or high-sucrose diets at 0.2% activated proliferator-activated receptor-gamma coactivator 1-α cascade and mitochondrial fusion, leading to the activation of mitochondrial biogenesis, and a thermogenic program [33]. Among others, they observed an increase in the nuclear respiratory factor-1 (Nrf1) gene expression in epididymal white adipose tissue, as well as a strong decrease in the size of this fat depot. Recently, Nrf1 was proposed in thermogenic fat cells as a metabolic guardian, avoiding tissue stress and inflammation independently of BAT differentiation, mass or expandability [34]. In addition, in a previous study we revealed that *Ilex paraguariensis*, rich in phenolic compounds, increased mitochondriogenesis and energy dissipation in brown adipose tissue and skeletal muscle, which explained the mass change of these oxidative tissues [35]. However, taking into account the lack of change in brown adipose tissue and gastrocnemius weights, and even more, in white adipose weights, it seems that under our experimental conditions, there was no sharp EE modification.

As far as the anti-inflammatory effect is concerned, no changes were found in the serum concentration of CRP. In the case of MCP1 and IL6, although serum concentrations were significantly reduced in rats fed the diets supplemented with the combination of W and CP when compared the MetS group, no significant changes were observed with rats fed the OC diet. Notwithstanding, a tendency toward a decrease was observed for the SAH rat group, which could become significant in a longer treatment. Effect of snack feeding on glycemic control was also evaluated. Although no clear improvement of glycemic control was found in rats treated with snack, lower NEFA values were detected for SAL, SAH, and SBH rat groups than MetS rats.

In addition, other parameters altered in MetS were also analyzed. After three weeks of treatment, the high dose of snack A reduced SBP. When we measured the activity of ACE, the enzyme that converts angiotensin I into angiotensin II, thus, increasing vasoconstriction, a reduction was observed in the SHA group, but only when it was compared with the OC group, not with the WC group. The SHB group showed a great increase. According to these results, which do not fit well with an anti-hypertensive action, it can be proposed that other mechanisms, not analyzed in the present work, are the basis for this effect. These results are in good accordance with those reported by Park et al. [36]. These authors observed a reduction in blood pressure in hypertensive rats induced via fucoidan obtained from W. This effect was due to increased eNOS activation by Akt phosphorylation, and thus increased NO production, to increased endothelium-dependent vasodilation, and to improved vascular elasticity.

We cannot state, given the amount of W and CP included in the snacks, that the amounts of their bioactive components reaching the cells are high enough to be effective, because unfortunately, no kinetic studies are available. This may be one of the reasons that explain the lack of anti-obesity and anti-inflammatory effects observed in MetS rats. Likewise, potential interactions among diet components can take place. Moreover, the thermal treatment needed to prepare the snacks can negatively affect the bioavailability and activity of CP and W bioactive components.

Our results cannot be compared with other studies because the snacks used in the present study have not been used previously. With regard to CP, no information is available concerning its potential effect on body fat. Nevertheless, we can consider reported studies carried out with W. In some of them, the algae showed an anti-obesity effect. In this sense, Park et al. (2011) observed a significant reduction in epididymal adipose tissue and adipocyte size in mice fed a high-fat diet supplemented with W [37]. According to this result and our in vitro data, an anti-obesity effect should have been found in our experiment in rats treated with the snacks. This apparent discrepancy could be due to the fact that mice have a faster TG metabolism in WAT than rats. However, this hypothesis is refuted by two facts. There are also studies in the literature that show reduced body weight and WAT weights in rats, and there are also reported results showing no anti-obesity effects in mice [21].

One might also consider the experimental period length, which was longer in the study reported by Park et al. (9 weeks) than in the present study (4 weeks) [37]. Giving support to this idea, Grasa-López et al. [15] reported decreases in WAT weight and adipocyte size in rats fed a high-fat diet supplemented with W at a dose of 400 mg/kg body weight for 8 weeks, as well as a reduction in serum inflammatory markers and blood pressure. Nevertheless, Kim et al. (2016) also showed a reduction in WAT weights by using W at concentrations from 0.6% to 1.7% (approximately 320 and 900 mg extract/kg body weight) during a shorter experimental period (5 weeks) [16]; consequently, it seems that this factor does not explain the discrepancy. It is important to point out that in the study reported by Kim et al., a reduction in energy intake, which should be related to a smaller WAT, was found, but a pair-fed group was not used in order to discard this confounding factor. The amounts of W used in the studies can also represent a differential factor. Regarding this issue, it should be emphasized that the amounts used in our study were lower (5–200 mg/kg body weight/day) than those used in the reported studies (320–900 mg/kg body weight/day). Due to the fact that our study, carried out in rats, was prior to a clinical intervention study that is now ongoing, we chose the doses of W to be included in the snack based on a sensory panel.

Another aspect related to the experimental design that should also be considered is that, in the above-mentioned studies, W was administered at the same time as the obesogenic diet; and thus the preventive effect of this algae was analyzed. By contrast, in our study, the snack containing W was included in the diet after inducing the MetS, and thus the treatment of this syndrome was assessed. This situation is not unique to W, it has also been observed by using, for instance, resveratrol. When the anti-obesity of this phenolic compound is analyzed, it can be observed that it is able to prevent fat accumulation associated to obesogenic diets, but not to reduce it when obesity has been previously established, or to increase fat reduction when it is administered in the frame of a hypocaloric diet [38]. Finally, it is important to point out that bearing in mind that final consumers for this type of snack would be adults, in our study adult rats were used. This fact differed from the majority of previous in vivo treatments, which were conducted with growing animals.

## 4. Materials and Methods

### 4.1. Ethical Approval

The animal experiment was approved in accordance with the Spanish regulation on animal experimentation (approval no M20_2017_003). All experimental protocols were reviewed and approved by the ethics committee on animal welfare of our institution (Comité Ético de Experimentación Animal de la Universidad del País Vasco, CEEA-UPV/EHU).

### 4.2. Reagents

Dimethyl sulfoxide, LPS from Escherichia coli O5:B55, penicillin/streptomycin (10,000 U/mL), and captopril were purchased from Sigma-Aldrich (St. Louis, MO, USA). Dulbecco’s modified Eagle’s medium (DMEM) was purchased from GIBCO (BRL Life Technologies, Grand Island, NY, USA). All other chemicals were obtained from Panreac Química S.A (Barcelona, Spain) unless otherwise stated.

### 4.3. Raw Material

CP was provided by Alimentaria Adín (Paterna, Valencia, Spain) and W seaweed was harvest and provided by Portomuiños (Galicia, Spain). Fresh raw materials were lyophilized and powdered via grinding and kept at −80 °C for further analysis.

### 4.4. Carob Pod and Wakame Extract Preparation

One gram of powdered W and CP was homogenized in 2 × 10 mL of 50% aqueous methanol via magnetic stirring for 1 h, and then centrifuged at 1635× *g* for 10 min at 4 °C. Supernatants of both methanolic extracts were mixed and filtered with filter paper (Whatman Grade 1). Using reduced pressure (SpeedVac Concentrator, Thermo Fisher Scientific Inc., Rockford, IL, USA) the obtained filtrates were evaporated and stored at −80 °C until analysis.

Methanolic extracts were used to evaluate bioactive properties, due to the fact that methanol has a higher dielectric constant than ethanol, which enables it to extract more polar (phenolic) compounds than ethanol.

### 4.5. In Vitro Assays: Anti-Hypertensive, Triacylglycerol-Lowering, and Anti-Inflammatory Activities

In order to conduct the ACE inhibitory activity, the method described by Shalaby et al. (2006) [39]. The fluorescence from release of Abz-Gly was determined at time zero by a microplate reader with excitation and emission wavelengths of 360 nm and 400 nm, respectively. Then, the assay plate was incubated at 37 °C for 30 min and the fluorescence was determined again. The obtained IC_50_ values were corrected for moisture and expressed as a gram of sample per liter of medium present during the reaction (g/L). The antihypertensive activity of captopril was used as a control of ACE inhibitor to evaluate the assay reproducibility. Experiments were carried out as independent duplicates assayed in triplicate.

3T3-L1 pre-adipocytes, supplied by American Type Culture Collection (Manassas, VA, USA) were cultured and stimulated to differentiate as previously reported [40]. From day 2 after differentiation, the medium contained fetal bovine serum /DMEM (10%) with 0.2 µg/mL insulin. This medium was changed every two days until the cell treatment (day 8). At day 8, >90% of cells developed mature adipocytes with visible lipid droplets. All medium contained 1% penicillin/streptomycin (10,000 U/mL), and the medium for differentiation and maturation contained 1% (*v/v*) of biotin and panthothenic acid. Cells were maintained at 37 °C in a humidified 5% CO_2_ atmosphere. For the treatment of mature adipocytes, first of all, preparation of extracts was carried out. W and CP methanolic extracts were dried in a vacuum concentrator (Speedvac, Eppendorf, Madrid, Spain), and subsequently, the dry weight was registered. For cell treatment, W and CP dry extracts were dissolved first in 95% ethanol and then in the treatment medium to prepare W (1 or 10 µg/mL) and CP (50 or 100 µg/mL) test solutions. The ethanol concentration in the medium never exceeded 0.1%. Cells grown in six-well plates were in expenditure cubated with either ethanol (control group) or with W, CP, and their combination at 1, 10, 50, and 100 µg/mL (diluted 95% ethanol) on day 8 after differentiation. The treatment medium contained only fetal bovine serum /DMEM (10%). After 24 h, the supernatant was collected, and cells were used for TG determination (Thermo Fisher Scientific Inc., Rockford, IL, USA) and glycerol quantification in the medium (Sigma, St. Louis, MO, USA) as described elsewhere [40]. Each experiment was performed three times.

The RAW 264.7 cell line (American Type Culture Collection, Rockville, MD, USA) from a mouse monocyte macrophage was used to study the anti-inflammatory activity of W, CP, or their combination. Cells were seeded in 96-well plates at a density of 2 × 10^4^ cells/well and maintained in DMEM supplemented with 10% FBS, and 1% penicillin/streptomycin in a humidified incubator containing 5% CO_2_ and 95% air at 37 °C as previously reported [17]. On the day before the assay, cells (80% confluent) were changed to an FBS-free medium to avoid potential interference of the serum. Dried W and CP extracts were dissolved in an FBS-free medium to prepare the W and CP test solutions. Cells were treated with W (1 or 10 µg/mL), CP (50 or 100 µg/mL), or their combination (W 10 µg/mL + CP 50 µg/mL, W 1 µg/mL + CP 50 µg/mL) for 24 h and sequentially elicited with 10 μg/mL of LPS from *Escherichia coli* O55:B5 for an additional period of 24 h. Cell treatments were performed in sextuplicate. Control wells, with cells in a culture medium without W and CP or LPS, were also included to observe potential effects of LPS elicitation and W and CP treatment. Finally, the medium was collected and NO production was quantified using the Griess reaction, following a previously described method [41]. Similarly, TNFα and PGD_2_ detection were carried out in the medium using a sandwich enzyme-linked immunosorbent assay (ELISA) (Diaclone SAS, Besancon Cedex, France, and Cayman Chemical, Ann Arbor, MI, USA).

### 4.6. RNA Extraction and Reverse Transcription Polymerase Chain Reaction (RT-PCR)

RNA samples from mature adipocytes and macrophages were extracted using Trizol (Invitrogen, Carlsbad, CA, USA), according to the manufacturer’s instructions. After RNA purity verification, samples were then treated with DNase I kit (Applied Biosystems, Foster City, CA, USA) to remove any contamination with genomic DNA. A total of 1.5 µg of RNA of each sample was reverse-transcribed to first-strand complementary DNA (cDNA) using iScriptTM cDNA Synthesis Kit (Bio-Rad, Hercules, CA, USA).

Relative mRNA levels of genes in adipocytes, as well as macrophages, were quantified using SYBR Green Master Mix or TaqMan^®^ Fast Advanced Master Mix (Applied Biosystems, Foster City, CA, USA) using Real-Time PCR with an CFX96 real-time PCR detection system (BioRad, Hercules, CA, USA). The sense/antisense primers (Metabion International AG, Steinkirchen, Germany) and annealing temperature in the case of SYBR Green RT-PCR are listed in Table 2. For Taqman technology, the following TaqMan^®^ Gene Expression Assays were used (Applied Biosystems, Foster City, CA, USA): *acca*, (mm01304285_m1); *fasn* (m00662319_m1); *pnpla2* (mm00503040_m1); *lipe* (mm00495359_m1); *slc2a4* (mm00436615_m1); *dgat1* (mm00515643_m1); and *dgat2* (mm499536_m1). The RT-PCR parameters used were those defined by manufacturers, with the exception of *slc42a*. In that case, RT-PCR consisted of fifty cycles of denaturation/annealing/extension. *β-actin* was used as the reference gene. All gene expression results were expressed as fold changes of the threshold cycle (Ct) value relative to controls using the 2^−ΔΔCt^ method [42].

### 4.7. Western Blot Analysis

Total protein was isolated from maturing 3T3-L1 adipocytes using 150 μL of lysis buffer (2 nM tris-HCl, 0.1 M sodium chloride, 1% Triton, 10% glycerol, 1 mM sodium orthovanadate, 2 mM EDTA, 1 mM phenylmethylsulfonyl fluoride, 2 mM sodium fluoride, and 1% protease inhibitor) and centrifuged (12,000× *g*, 15 min, 4 °C) to remove membranes and other proteic residues. Protein concentration was determined using a BCA protein assay kit (Thermo Fisher Scientific Inc., Rockford, IL, USA). Sixty micrograms of protein were subjected to 10% SDS polyacrylamide gel, electroblotted onto PVDF membranes (Merck KGaA, Darmstadt, Germany), and incubated with specific antibodies for FAS (1:250), β-actin (1:5000) (Santa-Cruz Biotech, Dallas, TX, USA) and Acetyl-CoA carboxylase enzyme (ACC; (1:1000) (Cell Signaling Technology, Danvers, MA, USA) overnight. Subsequently, polyclonal anti-mouse for *β-actin* and FAS, and anti-rabbit for ACC (1:5000) (Santa-Cruz Biotech, Dallas, TX, USA) were incubated for 2 h at room temperature, and FAS, ACC, and *β-actin* were measured. After antibody stripping, the membranes were blocked, and then incubated with phosphorylated ACC (serine 79, 1:1000) (Cell Signaling Technology, Danvers, MA, USA) antibodies. Bound antibodies were visualized by an ECL system (Thermo Fisher Scientific Inc., Rockford, IL, USA) and quantified using a Chemi-Doc MP imaging system (Bio-Rad, Hercules, CA, USA).

### 4.8. Fatty Acid Synthase Activity

FAS activity was measured with 75 μL of protein from a mature adipocyte homogenate using a spectrophotometer (Thermo Fisher Scientific Inc., Rockford, IL, USA) at 340 nm as previously reported [40].

### 4.9. Snack Preparation

A yeasted cracker based on a carob-seaweed-flour blend was formulated according to preliminary studies [17,24]. Dough samples were prepared by mixing baker’s yeast (0.48%), wheat (24.1%), oat (24.1%), CP (5.29% for snack A and 4.49% for snack B), and W flour (0.105% for snack A and 0.898% for snack B), carboxymethyl cellulose (0.289%), and extra olive oil (7.22%). Additional water was added as required to obtain easily handled doughs (38.5%). After mixing all ingredients for 5 min, the dough was allowed to rest at room temperature for 10 min and subsequently incubated for 40 min at 37 °C to allow the fermentation process to take place. Then, the doughs were laminated into a 2 mm thickness layer, cut into crackers (900 × 400 mm), and transferred to a baking sheet. Each batch was baked in a forced-air convection oven at 200 °C for 10 to 12 min. The final snack formulations were packed in modified atmosphere bags and stored at room temperature for the study. Protein, fat, moisture, ash, fiber, and carbohydrate contents were determined as detailed in Martín-Diana et al. [46]. Data were expressed as % of dry weight basis (d.w.b.).

### 4.10. Animals and Treatment Protocols

The experiment was conducted with 60 six-week-old male Wistar RccHan rats (203 ± 2.5 g) from Envigo (Barcelona, Spain). The rats were individually housed in polycarbonate metabolic cages (Tecniplast Gazzada, Buguggiate, Italy) and placed in an air-conditioned room (22 ± 2 °C) with a 12-h light–dark cycle. After a 6-day adaptation period, all rats were fed a high-fat, high-fructose diet (Brogardeen, Lynge, Denmark; Ref. D09100301) for eight weeks in order to develop MetS. This diet provided 40% of the energy as fat, 20% as protein, and 40% as carbohydrates where 20% was fructose (4.5 kcal/g diet). After this period, in order to establish the MetS baseline parameters, 10 fourteen-week old rats (MetS group) were sacrificed. The remaining 50 animals, fed a semi-purified, normal-caloric diet (3.9 kcal/g diet) for four weeks, were randomly divided into five experimental groups (*b* = 10): WC, OC, SAL, SAH, and SBH. The composition of each powder diet is described in Table 3. The WC diet represented the standard normal-caloric diet for the treatment of MetS in humans. It was prepared according to previous studies from our laboratory with adult rodents, as well as recommendations from the American Institute of Nutrition (AIN-93M) [47]. Considering that oat flour was highly present in the snack formulation, at the same level as wheat flour, starch for the OC diet came from oat and wheat flour in similar amounts. High-dose snack dietary interventions evaluated the effect of the complete substitution of complex carbohydrates with functional snacks [29]. The low-dose snack diet simulated a human intervention study, with an attainable functional snack intake (50 g per day, in a total contribution of complex carbohydrates of 250 g) [30].

At the end of the whole experimental period (8+4 weeks), eighteen-week old rats from the five experimental groups were sacrificed after 8–12 h of fasting, under anesthesia (chloral hydrate), via cardiac exsanguination. Serum was obtained from blood samples after centrifugation (1000 g for 10 min at 4 °C). Gastrocnemius muscle, IBAT, and WAT from different anatomical locations (epididymal, perirenal, mesenteric, and subcutaneous) were dissected, weighed, and immediately frozen. All samples were stored at −80 °C until further analysis.

### 4.11. Anti-Hypertensive, Anti-Inflammatory, and Glycemic Control Improvement Properties Evaluation of Functional Snack in MetS Rats

Blood pressure was recorded at the beginning and one week before the sacrifice, with the exception of the MetS group, whose blood pressure was measured at the end of the eight-week period used for MetS induction. SBP and DBP were recorded between 9 and 11 a.m. in rats using a non-invasive blood pressure system (LE50002, Panlab Harvard Apparatus, Barcelona, Spain). To minimize stress-induced variations in BP, all measurements were taken by the same person in the same peaceful environment and after acclimatizing animals to the procedure.

ACE activity was measured in the kidney by using a commercial kit (Spinreact, Girona, Spain). Briefly, 0.1 g of the right kidney was quickly homogenized in 2 mL of 50 mM Tris-HCl buffer, pH 7.4, containing 0.1% NaCl. Homogenates were centrifuged at 1000× *g* for 15 min at 4 °C and the supernatant was frozen at −20 °C. The protein contents of the samples were measured using the BCA method.

Specific rat ELISA kits were used for CRP (Biovendor, Brno, Czech Republic), monocyte chemotactic protein 1 (MCP1) (Thermo Fisher Scientific Inc., Rockford, IL, USA), interleukin-6 (IL6) (R&D Systems, Minneapolis, MN, USA) and insulin (Merck KGaA, Darmstadt, Germany) measurements in serum samples at the end of the experimental period. Glucose and NEFAs were measured using a commercially available spectrophotometer (Biosystems, Barcelona, Spain).

### 4.12. Statistical Analysis

Results were presented as mean ± standard error of the mean (SEM). After confirming the normal distribution of variables, differences among groups were determined using the Student’s *t*-test for the comparison of two groups or one-way analysis of variance (ANOVA), following Bonferroni’s multiple comparison using SPSS 24.0 software (SPSS Inc., Chicago, IL, USA). Statistical significance was defined as *p* < 0.05).

## 5. Conclusions

Taken as a whole, these results show that both W and CP show TG-lowering effects, but only the latter induced additional anti-inflammatory and anti-hypertensive actions. The combination of both components did not induce an additive or synergistic delipidating in vitro effect. As far as the in vivo study is concerned, snack B was ineffective, while snack A showed an anti-hypertensive effect in rats with MetS. However, the effects observed cannot be extrapolated to humans because this effect was only found with the highest dose, and so intervention studies should be addressed. Furthermore, due to the fact that statistical tendencies were observed in several parameters, further studies would be necessary to elucidate whether longer treatment periods could induce significant anti-obesity and anti-inflammatory effects.

## Figures and Tables

**Table 1:** Initial and final body weight, food and water intake, and white adipose tissue weights of MetS rats with treated experimental diets for 4 weeks.

	MetS *n* = 10	WC *n* = 10	OC *n* = 10	SAL *n* = 10	SAH *n* = 10	SBH *n* = 10
Food intake (g/day)	20.8 ± 0.5 ^b^	25.2 ± 1.4 ^a,b^	28.4 ± 1.3 ^a^	28.7 ± 1.2 ^a^	25.5 ± 1.0 ^a,b^	28.9 ± 1.5 ^a^
Energy intake (kJ/day)	391 ± 9 ^b^	418 ± 24 ^a,b^	471 ± 22 ^a^	473 ± 20 ^a,b^	409 ± 19 ^a,b^	463 ± 24 ^a,b^
Water intake (mL/day)	21.6 ± 0.9 ^b^	30.3 ± 0.2 ^a^	28.3 ± 1.1 ^a^	27.0 ± 1.1 ^a^	29.1 ± 0.7 ^a^	30.4 ± 1.9 ^a^
Initial body weight (g)	202 ± 2.5	202 ± 2.8	202 ± 2.5	203 ± 2.5	203 ± 2.6	204 ± 2.7
Final body weight (g)	411 ± 10.3	456 ± 13.8	449 ± 4.9	441 ± 10.5	441 ± 8.3	448 ± 11.8
White adipose tissue weights						
Epididymal (g)	11.7 ± 1.6	11.2 ± 1.2	12.2 ± 1.0	11.3 ± 0.8	11.7 ± 1.2	10.6 ± 1.2
Perirenal (g)	15.5 ± 1.4	16.1 ± 1.8	14.9 ± 1.0	14.5 ± 1.2	16.3 ± 1.5	14.8 ± 0.9
Mesenteric (g)	4.1 ± 0.4	5.0 ± 0.6	4.8 ± 0.3	5.2 ± 0.6	4.9 ± 0.4	4.4 ± 0.3
Subcutaneous (g)	14.1 ± 1.3	14.8 ± 1.6	13.9 ± 1.0	13.8 ± 1.0	14.0 ± 0.8	12.7 ± 0.4
∑ Adipose tissue (g)	45.4 ± 4.6	47.1 ± 4.9	45.8 ± 3.1	44.7 ± 3.5	46.9 ± 11.1	42.7 ± 2.9
∑ Adipose tissue/body weight (%)	10.9 ± 0.8	10.3 ± 0.9	10.2 ± 0.6	10.1 ± 0.7	9.4 ± 1.3	9.6 ± 0.7
IBAT weight (g)	1.46 ± 0.04	1.56 ± 0.10	1.40 ± 0.14	1.52 ± 0.08	1.57 ± 0.06	1.56 ± 0.08
∑ Gastrocnemius muscle weight (g)	4.58 ± 0.14	4.75 ± 0.16	4.18 ± 0.22	4.56 ± 0.13	4.90 ± 0.20	4.51 ± 0.26

IBAT: interscapular brown adipose tissue; MetS: Metabolic syndrome group; WC: wheat control group; OC: wheat-oat control group; SAL: the snack A low-dose group; SAH: snack A high-dose group; SBH: snack B high-dose group. Values are means ± SEM. Values in the same row with different subscripts are significantly different at *p* < 0.05.

**Table 2:** Primers for PCR amplification of each gene studied.

Primers	Sense Primer	Antisense Primer	Ref.	Annealing T(°C)
*β-actin*	5′-ACG AGG CCC AGA GCA AGA G-3′	5′-GGT GTG GTG CCA GAT CTT CTC-3′		60.0
*bax*	5′-GTG AGC GGC TGC TTG TCT-3′	5′-GTG GGG GTC CCG AAG TAG-3′		59.0
*bcl2*	5′-AGT ACC TGA ACC GGC ATC TG-3′	5′-GGG GCC ATA TAG TTC CAC AAA-3′		59.0
*cox2*	5′-GCT ACC TGG AGT ACA TGA AG-3′	5′-CTG TGA CTC CAG CTT ATC TG-3′	[43]	57.3
*il6*	5′-GCT ACC TGG AGT ACA TGA AG-3′	5′-GCA TAA CGC ACT AGG TTT GCC-3′	[44]	60.0
*nos2*	5′-CTT TGC CAC GGA CGA GAC-3′	5′-TCA TTG TAC TCT GAG GGC TGA C-3′		63.7
*scd1*	5′-TTC TTG CGA TAC ACT CTG GTG C-3′	5′-CGG GAT TGA ATG TTC TTG TCG T-3′	[45]	64.5
*tnfa*	5′-TCA CCT GCT GCT ACT CAT TC-3′	5′-TAC AGA AGT GCT TGA GGT GG-3′	[43]	62.7

*bax*, BCL2-associated X protein; *bcl2*, B cell leukemia/lymphoma 2; *cox2*, cyclooxygenase 2; *il6*, interleukin 6; *nos2*; nitric oxide synthase 2; *scd1*, stearoyl-Coenzyme A desaturase 1; *tnfα*, tumor necrosis factor α.

**Table 3:** Diet composition and suppliers.

Ingredients	WC (g/kg)	OC (g/kg)	SAL (g/kg)	SAH (g/kg)	SBH (g/kg)
Casein ^1^	200	200	200	200	200
Sucrose ^2^	230	230	230	230	230
Wheat starch ^3^	400	200	320	-	-
Oat starch ^3^	-	200	-	-	-
Snack A	-	-	80	400	-
Snack B	-	-	-	-	400
Sunflower oil ^2^	70	70	70	70	70
Cellulose ^3^	46	46	46	46	46
Mineral mix ^4^	35	35	35	35	35
Vitamin mix ^4^	11	11	11	11	11
l-methionine ^1^	4	4	4	4	4
Choline chlorhydrate ^1^	4	4	4	4	4

WC: wheat control group; OC: wheat-oat control group; SAL: the snack A low-dose group; SAH: snack A high-dose group; SBH: snack B high-dose group; ^1^ Sigma, St. Louis, MO, USA; ^2^ Local market; ^3^ Vencasser, Bilbao, Spain; ^4^ ICN Pharmaceuticals, Costa Mesa, CA, USA.
